# Plant Microfungi *Tranzschelia pruni-spinosae* and *Phragmidium rubi-idaei* Demonstrate Allergenic Capabilities in Mouse Models of Asthma

**DOI:** 10.3390/ijms27031507

**Published:** 2026-02-03

**Authors:** Piotr Wlaź, Katarzyna Socała, Magdalena Krasowska-Kunach, Marta Palusińska-Szysz, Urszula Świderska, Dominika Szczypior, Agnieszka Szuster-Ciesielska

**Affiliations:** 1 Biomedical Research Laboratory, Institute of Biological Sciences, Maria Curie-Sklodowska University, Akademicka 19 St, 20-033 Lublin, Poland; piotr.wlaz@mail.umcs.pl (P.W.); katarzyna.socala@mail.umcs.pl (K.S.); 2 Department of Virology and Immunology, Institute of Biological Sciences, Maria Curie-Sklodowska University, Akademicka 19 St, 20-033 Lublin, Poland; magda30krasowska@gmail.com (M.K.-K.); dominika.szczypior@gmail.com (D.S.); 3 Department of Genetics and Microbiology, Institute of Biological Sciences, Maria Curie-Sklodowska University, Akademicka 19 St, 20-033 Lublin, Poland; marta.palusinska-szysz@mail.umcs.pl; 4 Department of Botany, Mycology and Ecology, Institute of Biological Sciences, Maria Curie-Sklodowska University, Akademicka 19 St, 20-033 Lublin, Poland; urszula.swiderska@mail.umcs.pl

**Keywords:** fungal aeroallergens, IgE-mediated hypersensitivity, Th2 immune response, airway remodeling, plant pathogenic fungi, experimental allergenicity

## Abstract

Allergic conditions have surged to unprecedented levels globally, affecting approximately 30% of the global population. Fungi are among the most significant sources of allergens, accounting for approximately 6% of respiratory issues in the general population. However, identifying the precise cause of respiratory allergies remains challenging. We investigated the potential of two rust species, *Tranzschelia pruni-spinosae* and *Phragmidium rubi-idaei*, which infect common fruit plants, to induce inflammatory and asthmatic responses in mouse models of both acute and chronic asthma. Mice were sensitized and administered intranasal challenges with extracts from *T. pruni-spinosae* and *P. rubi-idaei*. Levels of pro-inflammatory cytokines (IL-4, IL-5, IL-13, TNF-α, and TGF-β) were measured via ELISA. Additionally, specific IgE production was assessed via ELISA and lung histology was examined using hematoxylin-eosin staining. Both fungal extracts induced significant increases in all tested cytokines, elevated specific IgE levels, and histological changes characteristic of acute and chronic asthma progression in the lungs. The microfungi *T. pruni-spinosae* and *P. rubi-idaei* possess strong proinflammatory and asthma-inducing capabilities, suggesting their potential as previously unrecognized fungal allergens.

## 1. Introduction

Allergic diseases are increasing worldwide, with the World Allergy Organization (WAO) reporting that 10–40% of individuals are affected, depending on the country. In many developed countries, more than 20% of the population experiences allergies [1,2]. At the beginning of the 20th century, allergies were relatively rare, but their prevalence has risen dramatically in recent decades. According to the European Academy of Allergy & Clinical Immunology (EAACI), over 150 million people in Europe suffer from chronic allergic diseases, with 20% of them experiencing severe and debilitating symptoms. It is anticipated that by 2025, allergies will affect half of the European population. These figures are probably underestimated, as many individuals either do not report their symptoms or are misdiagnosed [3,4].

Asthma is a major non-communicable disease affecting both children and adults. Although incidence and prevalence are higher among children, adults often face more serious morbidity and mortality [5]. According to the Australian Institute of Health and Welfare, asthma accounted for 35% of the overall burden of respiratory diseases in 2023 [6]. In 2022, the American Lung Association reported that 44.2 million people in the United States (13.5% of the population) had been diagnosed with asthma at some point, representing a 48% increase from 1999 (9.1%) [7]. Within the European Union (EU), asthma diagnoses in 2015 were recorded for 8.2% of adults and 9.4% of children [8]. The Global Asthma Report 2025 estimates that the number of asthma sufferers worldwide is 250–260 million [9].

Asthma is a chronic inflammatory condition characterized by variable airway constriction, leading to recurring episodes of coughing, wheezing, and chest tightness [10,11,12]. A key feature of asthma is airway obstruction due to narrowing of the bronchial tubes. This involves ongoing airway inflammation marked by immune cell infiltration and activation, including dendritic cells, eosinophils, neutrophils, various lymphocyte types, innate lymphoid cells, and mast cells. The complex interactions among these cells and the airway lining contribute to the development of asthma symptoms [13,14,15]. Airway hyperresponsiveness to diverse infectious and environmental triggers is a defining characteristic.

Fungal spores have been recognized as major inhalant allergens since the 12th century [16]. The precise prevalence of fungal allergies is not definitively known, but estimates suggest that 3% to 10% of the general population may be affected, depending on regional climatic conditions [17]. The best-documented allergenic fungi belong to the genera *Cladosporium, Penicillium*, *Aspergillus*, and *Alternaria* [18]. *Cladosporium herbarum* and *Alternaria alternata* rank as the third most common inhalant allergens, following house dust and grass pollen, underscoring the need to evaluate the risks posed by airborne fungal spores [19]. Fungal spores can trigger IgE-mediated type I hypersensitivity reactions, including allergic rhinitis and asthma. Elevated airborne spore levels correlate with increased hospital visits and asthma-related mortality, as well as a higher risk of rhinitis [17,20]. Fungal-related respiratory conditions also include bronchopulmonary mycoses, allergic sinusitis, and hypersensitivity pneumonitis [21,22]. Standard skin or blood tests often fail to identify the specific allergen responsible, indicating the existence of unrecognized allergens. In our previous studies using models of the upper (BEAS-2B cell line) and lower (A549 cell line) respiratory tract, we first demonstrated that microfungi parasitizing common fruit plants such as *Prunus domestica* and *Rubus idaeus*—specifically, *T. pruni-spinosae* and *P. rubi-idaei*—exhibited proinflammatory and proallergenic effects [23]. The current study aimed to evaluate whether extracts from *T. pruni-spinosae* and *P. rubi-idaei* induce inflammatory and asthmatic responses in acute and chronic mouse models, assessing cytokine production, specific IgE levels, and lung tissue changes.

## 2. Results

### 2.1. Composition of the Peripheral Blood Leukocytes in the Examined Mice

In the acute asthma model, significant differences were observed in all leukocyte subpopulations (one-way ANOVA: df = 4, 35 for all analyses), including lymphocytes (F = 144.9, *p* < 0.0001), granulocytes (F = 195.3, *p* < 0.0001), eosinophils (F = 4.63, *p* < 0.0001), and monocytes (F = 15.86, *p* = 0.0042). In the chronic asthma model, significant differences (one-way ANOVA: df = 4, 35 for all analyses) were found in lymphocyte (F = 103.8, *p* < 0.0001), granulocyte (F = 252.5, *p* < 0.0001), and eosinophil (F = 8.49, *p* < 0.0001) percentages, whereas monocyte levels remained unchanged across groups (F = 0.909, *p* = 0.470).

**Figure 1:**
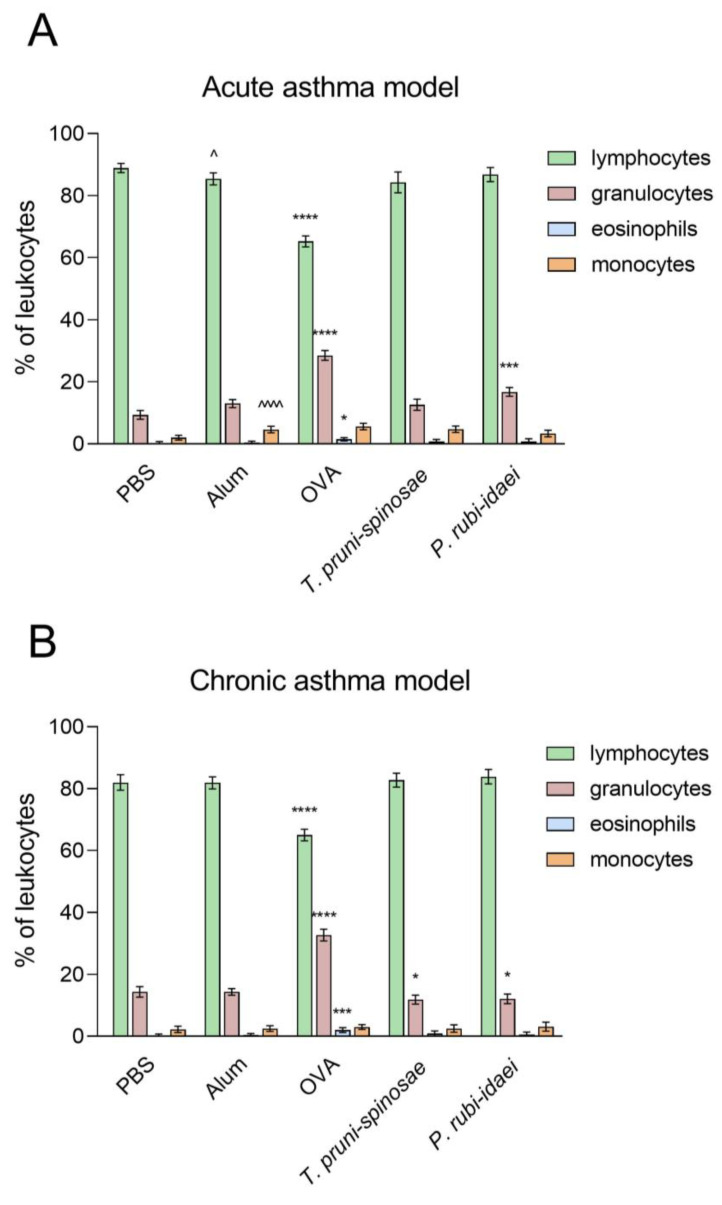
Percentage of individual groups of peripheral blood leukocytes in the mice studied: (**A**)—acute asthma model, (**B**) chronic asthma model. PBS—negative control, Alum—adjuvant control (Al(OH)_3_), OVA—positive control (ovalbumin). Data are presented as means ± SD. * *p* < 0.05, *** *p* < 0.001, and **** *p* < 0.0001—statistically significant differences compared to the adjuvant control (Alum); ^ *p* < 0.05 and ^^^^ *p* < 0.0001—statistically significant differences compared to the negative control group (PBS) (one-way ANOVA with the Tukey post hoc test).

In the adjuvant control group, mice had lymphocyte percentages of 85.4 ± 1.92 and 81.9 ± 1.96 in the acute and chronic asthma models, respectively. This was significantly reduced only in the ovalbumin-treated group, both in the acute (65.25 ± 1.75) and chronic (65.0 ± 1.85) asthma models (*p* < 0.0001). Conversely, treatment with extracts from *T. pruni-spinosae* and *P. rubi-idaei* did not cause significant changes in lymphocyte percentages. In addition, there was a slight, though statistically significant (*p* < 0.05) reduction of lymphocytes in the adjuvant control group compared with the PBS control group (Figure 1A,B).

In the acute asthma model, the percentage of blood granulocytes in the ovalbumin group (28.5 ± 1.6; *p* < 0.0001) and the *P. rubi-idaei* extract group (16.75 ± 1.39; *p* < 0.001) was significantly higher than in mice from the adjuvant group (13.0 ± 1.31). Conversely, after intranasal inhalation of *T. pruni-spinosae* extract, the blood granulocyte counts were similar to those in the adjuvant-treated group (12.6 ± 1.77). In the chronic asthma model, the percentage of blood granulocytes was significantly increased in the ovalbumin-treated group (32.63 ± 1.92; *p* < 0.0001). In contrast, treatment with *T. pruni-spinosae* (11.88 ± 1.46) and *P. rubi-idaei* (12.13 ± 1.55) extracts resulted in a slight but statistically significant (*p* < 0.05) decrease in granulocyte counts as compared to the adjuvant control group (14.38 ± 1.06) (Figure 1A,B).

**Figure 2:**
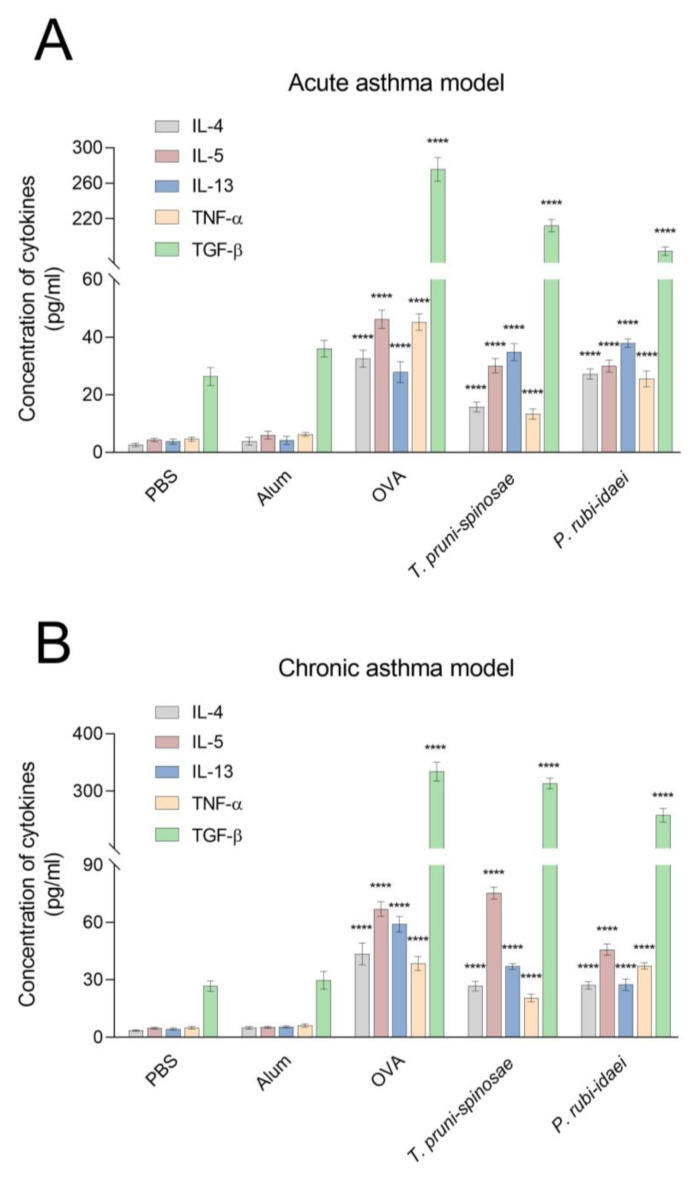
Levels of cytokines in mouse sera in acute (**A**) and chronic (**B**) asthma models. PBS—negative control, Alum—adjuvant control (Al(OH)_3_), OVA—positive control (ovalbumin). Data are presented as means ± SD. **** *p* < 0.0001—statistically significant differences compared to the adjuvant control group (Alum) (one-way ANOVA with the Tukey post hoc test).

In both the acute and chronic asthma models, a significant increase in eosinophil percentage was observed only in the ovalbumin-treated group (1.5 ± 0.54, *p* < 0.05 and 2.0 ± 0.76, *p* < 0.001 in acute and chronic asthma models, respectively) as compared to the adjuvant control group (0.38 ± 0.52 in both acute and chronic asthma models) (Figure 2A,B). In the acute asthma model, a statistically significant difference (*p* < 0.0001) in monocyte percentage was observed between the negative control group (2.00 ± 0.76) and the adjuvant control group (4.63 ± 1.06). After chronic asthma induction, the proportion of monocytes remained consistent across all studied groups. Overall, no notable differences were observed between acute and chronic asthma induction concerning blood leukocyte subpopulations.

### 2.2. Cytokine Levels in Mouse Sera Following the Induction of Acute and Chronic Asthma

One-way ANOVA revealed statistically significant changes in serum levels of IL-4 (F = 421, *p* < 0.0001), IL-5 (F = 569, *p* < 0.0001), IL-13 (F = 404, *p* < 0.0001), TNF-α (F = 571, *p* < 0.0001), and TGF-β (F = 1803, *p* < 0.0001) in the acute asthma model (df = 4, 35 for all analyses). Specifically, ovalbumin challenge (32.5 ± 2.98 pg/mL) induced an approximately 8-fold increase in IL-4 levels compared with the adjuvant control group (3.88 ± 1.36 pg/mL). A comparable increase was observed in the *P. rubi-idaei*–treated group (27.22 ± 1.78 pg/mL), while the increase following treatment with *T. pruni-spinosae* (15.77 ± 1.71 pg/mL) was relatively lower than in ovalbumin and *P. rubi-idaei* groups. Similarly, ovalbumin exposure (46.25 ± 3.20 pg/mL) resulted in an 8-fold increase in IL-5 concentration compared with the adjuvant control group (6.00 ± 1.31 pg/mL). Both *T. pruni-spinosae* and *P. rubi-idaei* induced 5-fold increases in IL-5 (30.09 ± 2.45 and 29.99 ± 2.05 pg/mL, respectively). Serum IL-13 concentration increased from 4.23 ± 1.42 pg/mL in the adjuvant control group to 27.88 ± 3.68 pg/mL in the ovalbumin-treated group. *T. pruni-spinosae* and *P. rubi-idaei* caused a further increase in IL-13 (to 34.87 ± 2.93 and 37.88 ± 3.68 pg/mL, respectively). Both fungal extracts had relatively weaker effects on serum TNF-α concentrations (37.88 ± 1.73 pg/mL for *T. pruni-spinosae* and 25.53 ± 2.73 pg/mL for *P. rubi-idaei*) compared with the elevation caused by ovalbumin (45.25 ± 2.92 pg/mL vs. 6.31 ± 0.66 pg/mL in the adjuvant control group). Likewise, TGF-β showed the highest concentration in ovalbumin-treated mice (275.6 ± 3.41 pg/mL vs. 36.04 ± 2.93 pg/mL in the adjuvant control group), while *T. pruni-spinosae* and *P. rubi-idaei* increased their levels to 212.30 ± 7.13 and 183.50 ± 4.78 pg/mL, respectively (Figure 2A; Tukey post hoc test: *p* < 0.0001 for all comparisons between the adjuvant group and other treatment groups).

In the chronic asthma model, statistically significant changes (one-way ANOVA: df = 4, 35 unless otherwise stated) were also reported for all measured cytokines: IL-4 (F = 265, *p* < 0.0001), IL-5 (F = 1336, *p* < 0.0001), IL-13 (F(4,34) = 713, *p* < 0.0001), TNF-α (F = 479, *p* < 0.0001), and TGF-β (F = 1759, *p* <0.0001). Ovalbumin challenge caused a marked increase in all measured cytokines compared with the adjuvant control group. IL-4 concentration increased from 4.90 ± 0.68 pg/mL in the adjuvant control group to 43.50 ± 5.73 pg/mL in ovalbumin group. In mice treated with *T. pruni-spinosae* (26.72 ± 2.46 pg/mL) and *P. rubi-idaei* (27.10 ± 1.86 pg/mL), IL-4 levels were significantly elevated, but the increase was lower than in the ovalbumin group. Serum IL-5 level increased from 5.23 ± 0.61 pg/mL in control adjuvant group to 67.00 ± 3.82 pg/mL in ovalbumin-treated mice. The highest IL-5 level was found in *T. pruni-spinosae* group (75.33 ± 3.14 pg/mL), while *P. rubi-idaei* produced a smaller increase (45.74 ± 2.88 pg/mL). Ovalbumin exposure (59.00 ± 4.04 pg/mL) caused a 10-fold elevation in IL-13 concentration compared with the adjuvant control group (5.43 ± 0.52 pg/mL). The increase in serum IL-13 in *T. pruni-spinosae* and *P. rubi-idaei* groups was relatively lower than in ovalbumin group (36.91 ± 1.49 pg/mL and 27.47 ± 2.95 pg/mL, respectively). TNF-α levels increased from 6.15 ± 0.86 pg/mL in the control adjuvant group to 38.38 ± 3.70 pg/mL following ovalbumin exposure. *P. rubi-idaei* caused a similar effect, increasing TNF-α level to 37.18 ± 1.67 pg/mL, while the increase in *the T. pruni-spinosae* group (20.52 ± 2.03 pg/mL) was relatively lower than in ovalbumin and *P. rubi-idaei* groups. TGF-β showed the highest concentration among all cytokines, increasing from 29.75 ± 4.53 pg/mL in the adjuvant control group to 333.80 ± 16.58 pg/mL after ovalbumin challenge. In mice receiving *T. pruni-spinosae* and *P. rubi-idaei*, TGF-β levels increased to 313.30 ± 9.38 pg/mL and 258.10 ± 11.73 pg/mL, respectively (Figure 2B).

### 2.3. Serum Levels of Allergen-Specific IgE in Mice After the Induction of Acute and Chronic Asthma

**Figure 3:**
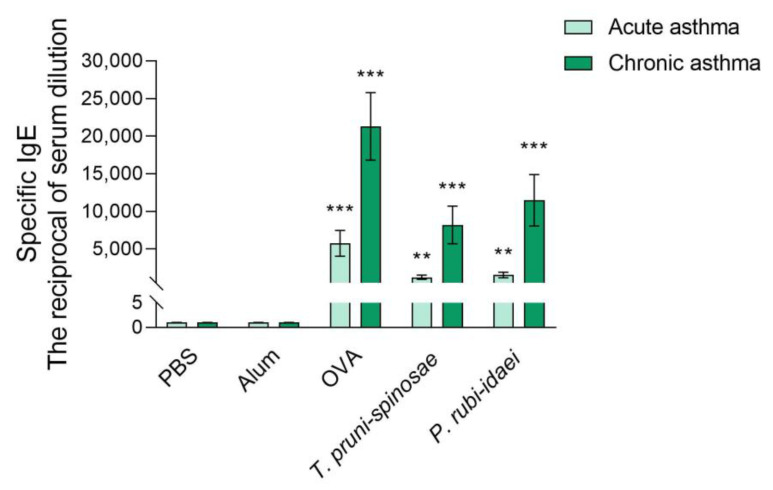
Levels of mouse serum IgE antibodies in acute and chronic asthma models. PBS—negative control, Alum—adjuvant control (Al(OH)_3_), OVA—positive control (ovalbumin). Data are presented as means ± SD. ** *p* < 0.01, *** *p* < 0.001—statistically significant difference compared to the adjuvant control group (Alum) (Kruskal–Wallis test followed by the Dunn’s multiple comparison test).

In both acute and chronic asthma models, a statistically significant increase in specific IgE antibodies targeting ovalbumin and proteins from *T. pruni-spinosae* and *P. rubi-idaei* extracts was observed (*p* < 0.02 in the acute asthma model and *p* < 0.02 in the chronic asthma model, as determined by the Kruskal–Wallis test). However, the extracts from *T. pruni-spinosae* and *P. rubi-idaei* were much less effective than ovalbumin in eliciting specific IgE responses (Figure 3). As IL-4 plays a role in IgE production, Spearman correlation analysis between IgE and IL-4 levels was performed. A strong positive correlation during chronic asthma development was observed for all three allergens: OVA (r = 0.88, *p* < 0.05), *T. pruni-spinosae* (r = 0.94, *p* < 0.05), and *P. rubi-idaei* (r = 0.9, *p* < 0.05).

### 2.4. Histological Analyses of Mouse Lungs

**Figure 4:**
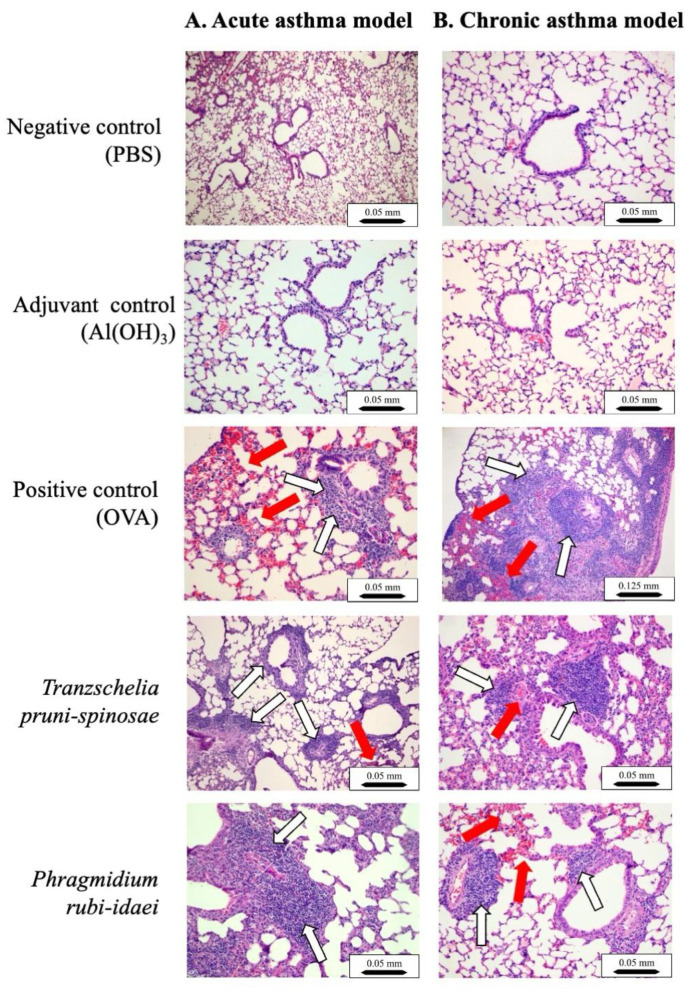
Anatomical and structural lung lesions in mice after sensitization and asthma challenges. Lung sections were stained with H&E after paraffin embedding and examined under a light microscope—Panel (**A**) illustrates the acute asthma model, while Panel (**B**) shows the chronic asthma model. The lung tissue displayed focal eosinophil recruitment (red arrows), along with lymphocytes, macrophages, and neutrophils (white arrows). Scale bar: 0.05 mm (×10 magnification) or 0.125 mm (×4 magnification).

In both the negative and adjuvant control groups, the microscopic lung structure remained normal, regardless of whether the asthma was acute or chronic (Figure 4A,B). In the positive control group (OVA) of the acute asthma mouse model, dense inflammatory infiltrates were observed around bronchi, blood vessels, and peribronchiolar regions. These infiltrates mainly consisted of neutrophils and eosinophils, along with some lymphocytes and macrophages. Five animals showed focal interstitial pneumonia, mostly located in the central (parabronchial) lung area. Mild hyperplasia was observed in the bronchial and bronchiolar epithelial layers. Congestion and focal interalveolar hemorrhages were also observed (Figure 4A).

Similar multifocal inflammatory infiltrates were observed in the positive control (OVA) within the chronic asthma mouse model, although their composition slightly differed, mainly featuring plasma cells and lymphocytes. Eosinophils and neutrophils remained present. More widespread focal pneumonia affected seven animals and was located in the same region as in the acute group. Foamy macrophages formed focal interalveolar clusters. Small lymphocyte accumulations near the pleura were observed, particularly in two mice. Eight animals showed severe thickening of the walls of small blood vessels. Mild hyperplasia of the bronchiolar epithelium was present. Congestion and localized interalveolar hemorrhages were also noted (Figure 4B).

Following sensitization and intranasal challenge with *T. pruni-spinosae* extracts in the acute asthma model, lung lesions resembled those observed in the positive control (OVA) group but were milder. One mouse exhibited more extensive pneumonia (Figure 4A). In the chronic asthma group, lung lesions were similar to those in the positive control but less severe, and one mouse again showed extensive pneumonia. In both models, lungs displayed an accumulation of eosinophils, lymphocytes, and granulocytes (Figure 4A,B).

In the acute asthma model, mice intranasally sensitized and challenged with *P. rubi-idaei* extract exhibited responses similar to those induced by ovalbumin and *T. pruni-spinosae*, including marked leukocyte infiltration. Two cases showed more severe pneumonia (Figure 4A). Lung tissue analysis from these mice revealed lesions comparable in nature and severity to those observed in the positive control (OVA) group (Figure 4B).

## 3. Discussion

Allergic conditions are considered a major public health concern of the 21st century in developed countries, affecting over 30% of the population and with rapidly rising prevalence. Fungi are the most significant source of allergens among the options. Identified fungal allergens include 174 from the Ascomycota phylum and 30 from the Basidiomycota phylum. The main allergenic fungi belong to the genera *Alternaria*, *Aspergillus*, *Cladosporium*, *Penicillium*, and *Fusarium* [24,25]. Patients are often not explicitly diagnosed with the specific allergens to which they are sensitive. Therefore, we present for the first time two phytopathogenic microfungi—rust fungi from the Pucciniales order, specifically *T. pruni-spinosae* and *P. rubi-idaei*—that exhibit allergenic potential in murine models of acute and chronic asthma. These findings strongly support our earlier research, which used in vitro models of the upper (BEAS-2B cell line) and lower (A549 cell line) respiratory tracts, in which *T. pruni-spinosae* and *P. rubi-idaei* demonstrated pro-inflammatory and pro-allergenic effects [23].

The Global Asthma Report states that asthma affects 9.1% of children, 11.0% of adolescents, and 6.6% of adults worldwide. This respiratory condition has a significant impact on patients and their families worldwide, creating substantial psychological, medical, and financial burdens [26]. Besides genetic factors, the root cause of asthma is the inflammatory response in the respiratory system to normally harmless environmental substances, whether inorganic or organic. Chronic inflammation and the clinical manifestations of asthma develop when interactions between inflammatory cells and resident cells initiate a cascade of events [5,10,27]. Given the crucial role of white blood cells in immune and inflammatory responses, we examined the differences in specific leukocyte populations in the blood and lungs of mice with acute or chronic asthma compared with healthy controls.

Research consistently demonstrates that higher levels of lymphocytes and eosinophils are distributed throughout the airways in asthma patients, regardless of the disease severity. Furthermore, eosinophil accumulation might be associated with the severity of asthma symptoms [15,28,29,30]. In our study using an acute model, we observed a significant reduction in blood lymphocytes after administration of ovalbumin and two fungal extracts. This suggests that these white blood cells migrate to the respiratory system, consistent with how the allergens were delivered. Analysis of lung samples from allergic mice showed an influx of lymphocytes and eosinophils, especially when allergens were administered over a prolonged period. Although eosinophilic inflammation is typical of asthma development, neutrophilic inflammation also plays a key role. Sometimes, both eosinophils and neutrophils are present simultaneously. Asthma is classified into three types based on the dominant cell types in sputum: eosinophilic asthma, neutrophilic asthma, and mixed neutrophilic–eosinophilic asthma [13,31]. Our histological analysis qualitatively confirmed the presence of lymphocytes and eosinophils in peribronchial and perivascular regions in both asthma models, but their distribution was not quantitatively assessed.

In our study, blood neutrophil levels increased only in response to the classical asthma inducer, ovalbumin, in both acute and chronic disease models. Following sensitization with *T. pruni-spinosae* and *P. rubi-idaei*, blood neutrophil percentages remained similar to those in the adjuvant control group. However, it is possible that neutrophils migrated from the blood to the lungs, as confirmed by histological analysis in both the acute and chronic asthma models. In contrast, a significant infiltration of eosinophils in both blood and lungs was observed after sensitization with either fungus, similar to the positive control (OVA).

Different fungal species facilitate the migration of eosinophils, lymphocytes, and neutrophils into the lungs. Lilly et al. reported that in an experimental model of fungal asthma, mice repeatedly exposed to *A. fumigatus* exhibited a predominance of eosinophils in their lungs. The study also observed that during acute *A. fumigatus* challenges, neutrophils became the main cell type present in the lungs [28]. Janssens et al. further corroborated the influx of eosinophils and neutrophils into lungs in a murine model following *A. fumigatus* exposure [32]. In a different allergy mouse model triggered by *A. alternata* and *Cladosporium herbarum*, researchers noted pulmonary inflammation marked by elevated lung neutrophils, eosinophils, and lymphocytes [33].

In mouse studies, injecting OVA—used as a model antigen—mixed with aluminum hydroxide (an adjuvant that promotes Th2 cell development) into the peritoneal cavity produced OVA-specific Th2 cells. It is important to note that the IP sensitization route with adjuvant, although not physiologically typical, is widely employed in the field [34,35,36]. These cells produced IL-4, IL-5, IL-10, and IL-13 along with antigen-specific IgE [37]. We also used an OVA antigen model to compare the activity of *T. pruni-spinosae* and *P. rubi-idaei*. Both fungal extracts significantly elevated IL-4, IL-5, and IL-13 levels in mice with acute and chronic asthma. However, cytokine measurements were limited to blood samples and did not include the disease site. Compared to the acute asthma model, prolonged exposure to all allergens (OVA, *T. pruni-spinosae*, and *P. rubi-idaei*) resulted in a significant increase in IL-5 and TGF-β levels. Furthermore, during chronic asthma induction, serum IL-4 levels in mice also showed a notable rise.

Research shows that TNF-α, elevated in severe asthma cases, contributes to barrier dysfunction and activates cells in bronchial epithelial tissue [38]. Elevated levels of this cytokine are associated with increased reactive oxygen species (ROS) production, which damages the connections between bronchial epithelial cells by decreasing E-cadherin and occludin expression. Our lab experiments clearly showed that *T. pruni-spinosae* and *P. rubi-idaei* significantly increase TNF-α and ROS levels in models of both the upper (BEAS-2B cell line) and lower (A549 cell line) respiratory tracts. This rise was positively linked to the disruption of epithelial cell junctions, as indicated by decreased E-cadherin and occludin [23]. During the acute phase of asthma, inhalation of *T. pruni-spinosae* and *P. rubi-idaei* resulted in lower TNF-α levels than in the ovalbumin group. Yet, in the chronic asthma mouse model, serum cytokine levels were similar across all allergen-exposed groups. Research indicates that transforming growth factor beta (TGF-β) and Th2 cytokines (IL-5 and IL-13) are involved in airway remodeling as asthma progresses [39,40]. Numerous investigations have noted elevated TGF-β activity in asthmatic conditions [41,42]. TGF-β affects airway remodeling via several pathways, including promoting the multiplication of airway smooth muscle (ASM) cells and inducing epithelial cell death. Additionally, TGF-β has strong profibrotic effects on mesenchymal cells. Studies with an allergic asthma animal model have shown that this growth factor boosts extracellular matrix deposition, encourages ASM cell proliferation, and elevates mucus production [43,44]. Namvar et al.’s research showed that *A. fumigatus* conidia (strain Af293) induced the production of key profibrogenic growth factors, notably TGF-β1 and TGF-β2, in primary airway epithelial cells [45]. Additionally, mice exposed to *A. fumigatus* spores showed elevated TGF-β levels, which correlated with significant inflammation and airway remodeling [46]. Our studies observed notably elevated levels of TGF-β after intranasal exposure to the three allergens (OVA, *T. pruni-spinosae*, and *P. rubi-idaei*). Furthermore, compared to acute asthma, the cytokine levels were substantially higher during the chronic phase of the disease. However, it’s important to note that since TGF-β needs activation outside the cell for activity, the TGF-β detected in our research might not directly indicate its functional activity.

The role of IgE antibodies in causing allergic conditions, such as asthma, has been well documented. During the classic immediate hypersensitivity response, allergens with multiple binding sites cross-link IgE antibodies attached to mast cells via the high-affinity IgE receptor (FcϵRI). This triggers the release of stored vasoactive substances, activates cytokine gene transcription, and stimulates the production of new prostaglandins and leukotrienes. In the respiratory tract, these chemicals quickly cause mucosal swelling in the bronchi, increase mucus secretion, and lead to smooth muscle contraction. These processes also attract inflammatory cells to the affected area [47,48]. Fungal components can act as allergens, causing an IgE-mediated immune response that plays a role in both the onset and progression of asthma [25,49]. For instance, elevated levels of specific IgE were observed in asthma patients, where *A. fumigatus* was identified as a causative factor [50,51].

Additionally, studies demonstrated that IgE levels strongly correlated with IL-4 production following *A. fumigatus* exposure in both mice [52,53] and humans [54]. Extracts from *T. pruni-spinosae* and *P. rubi-idaei* effectively stimulated specific IgE production in both acute and chronic asthma mouse models. However, these IgE levels were significantly lower than those observed after intranasal ovalbumin exposure. Furthermore, there was a strong positive correlation between IL-4 production and blood IgE levels following exposure to the three allergens in the chronic asthma mouse model.

Interestingly, despite clear induction of extract-specific IgE in both asthma models, the absolute titers remained significantly lower than those elicited by intranasal OVA. This discrepancy likely reflects both quantitative and qualitative differences between the antigens. Unlike the single, highly soluble protein OVA, the crude rust fungal extracts contain a complex mixture of proteins, glycoproteins, and cell-wall components (e.g., β-glucans, chitin, mannans) that may be less efficient in driving a strong classical Th2/IgE response, while simultaneously providing potent innate immune stimuli through pattern-recognition receptors on epithelial and myeloid cells. Thus, the relatively low specific IgE titers, in the presence of marked airway inflammation and histopathological changes, suggests that non-IgE-mediated mechanisms—including direct innate immune activation and potentially Th17-skewed responses—make an important contribution to the asthma-like reaction induced by these rust fungi. Although this study was not designed to dissect these pathways in detail, these findings indicate that IgE represents only one component of the complex immune response to environmental rust fungi and warrant further mechanistic studies.

The airway epithelium functions as a complex barrier that provides physical, functional, and immune protection against inhaled environmental particles. This defense is essential for preserving health. However, when the barrier is damaged, it can trigger immune and inflammatory responses to external allergens, microbes, and pollutants. Such vulnerability may lead to chronic inflammatory diseases like allergic rhinitis, chronic rhinosinusitis, and asthma. In these upper airway conditions and asthma, the epithelium becomes dysfunctional due to disrupted formation of tight junctions involving E-cadherin and occludin [29,45,46,55,56]. In fact, during the development of asthma, airway remodeling begins with epithelial changes, including shedding, destruction of ciliated cells, and epithelial cell hyperplasia [57].

Airway remodeling plays a vital role in the clinical presentation of chronic asthma. It involves structural changes in both large and small airways, impacting cellular elements and the extracellular matrix. The remodeling process includes epithelial cell death, increased proliferation of airway smooth muscle cells, and fibroblast activation. These structural modifications within the airway wall and submucosa are driven by complex interactions among various cell types, such as eosinophils, neutrophils, and lymphocytes. These pathological changes collectively influence the progression and symptoms of chronic asthma [57,58,59].

Labram et al. found that both human and murine airway epithelial cells exposed to A. fumigatus spores secrete pro-fibrogenic factors like TGF-β1, TGF-β2, periostin, and endothelin-1. These factors lead to significant inflammation and airway remodeling, evidenced by epithelial denudation, subepithelial fibrosis with more extracellular matrix, extensive smooth muscle hypertrophy, and goblet cell hyperplasia [46].

Our study examined inflammatory symptoms in mice lungs after inhaling fungal extracts from *T. pruni-spinosae* and *P. rubi-idaei*. Histological analysis revealed changes similar to those caused by ovalbumin, though less severe in both acute and chronic asthma models. We observed dense inflammatory infiltrates of neutrophils and eosinophils in multifocal peribronchiolar, peribronchial, and perivascular regions, along with mild hyperplasia of bronchial and bronchiolar epithelial layers, and significant thickening of small vessel walls. While extensive focal pneumonia was noted in the OVA-positive group, individual cases of pneumonia were also present in the groups treated with *T. pruni-spinosae* and *P. rubi-idaei.* Our data suggest that the modulation of systemic cytokines by the extracts may be compatible with improved epithelial barrier function, although direct markers of barrier integrity were not assessed in this study.

## 4. Materials and Methods

### 4.1. Plant Material and Morphological Identification

The study focused on biotrophic fungi that cannot be cultivated on artificial media in laboratory settings. Therefore, research specimens were collected from their natural habitats. Rust species (Pucciniales) impacting plant organs were gathered in Rymanów, Poland. *T. pruni-spinosae* samples were obtained from *Prunus domestica* L. on 11–12 September 2021, and 1–2 October 2021 (LBL M–033122, leg. U. Świderska). *P. rubi-idaei* specimens were collected from *Rubus idaeus* L. on the same dates (LBL M–033123, leg. U. Świderska). These host plants are frequently found in forests and thickets (*Rubus idaeus* L.) or are cultivated by growers (*Rubus idaeus* L., *Prunus domestica* L.). They are not protected species and are located outside protected areas.

The specimens, which include leaves and their fungal pathogens, were air-dried and stored in the herbarium at Maria Curie-Skłodowska University in Lublin (LBL). U. Świderska, a mycologist, initially examined the samples’ morphological features by preparing microscopic slides stained with Lactophenol Cotton Blue and observing them under an Olympus BX53 light microscope at 40×, 100×, 400×, and 600× magnifications. An Olympus digital camera SC180 was attached to capture images, while an Olympus SZ10 stereoscopic microscope with an Olympus XC50 camera (Olympus, Tokyo, Japan) was used for microphotography of the fungal structures. Additionally, some structures were coated with gold using an Emitech K550X Sputter Coater (Quorum Technologies Ltd. (Quorum Emitech), Ashford, Kent, UK) and examined with a TESCAN Vega 3 LMU scanning electron microscope (SEM) (TESCAN Brno, s.r.o., Brno, Czech Republic). Samples containing teliospores of the studied fungal species were further prepared for laboratory analysis using an Olympus SZ61 stereoscopic microscope (Olympus, Tokyo, Japan). The taxonomic classification of *T. pruni-spinosae* and *P. rubi-idaei* was confirmed in a previous genetic study [23]. The collected material was placed in test tubes, exposed to liquid nitrogen vapor for 24 h, then ground with a mortar and pestle. The resulting fungal powder was used to prepare crude extracts.

### 4.2. Preparation of Crude Fungal Extracts

The fungal samples underwent three acetone washes, followed by dehydration at 37 °C for 24 h. After drying, samples were suspended in 0.05 M Tris-HCl buffer (pH 8.0) using 1 mL of buffer per 10 mg of dry weight. The mixture was sonicated three times in a room-temperature water bath (Elmasonic S100H, Elma, Singen, Germany), with each session lasting 20 s and followed by 2 min of cooling. The extraction was then carried out overnight with gentle agitation at 4 °C. Subsequently, the extracts were centrifuged at 804× *g* for 10 min at 4 °C. The supernatants were transferred to a regenerated cellulose membrane with a 6–8 kDa MW cutoff (Spectrum Laboratories, Rancho Dominguez, CA, USA) and dialyzed against 0.1M NH_4_HCO_3_ buffer (pH 8.4) for 24 h at 4 °C, with the buffer changed three times. After dialysis, the samples were lyophilized and reconstituted in phosphate-buffered saline (PBS, Biomed, Lublin, Poland). Protein concentrations were determined using the Pierce BCA Protein Assay Kit (Thermo Fisher Scientific, Waltham, MA, USA), with bovine serum albumin as the standard [24]. The crude fungal extracts, prepared in triplicate, were divided into 100 μL aliquots and stored at −80 °C for future analyses.

### 4.3. Animal Model and Experimental Design

#### 4.3.1. Animals

The study involved adult female BALB/cmbd mice, aged 4–6 weeks, sourced from the Center for Experimental Medicine at the Medical University in Białystok, Poland. The animals were housed in the animal facility of the Biomedical Research Laboratory, an accredited unit at the Institute of Biological Sciences at Maria Curie-Skłodowska University in Lublin, Poland. Before the experiments, the mice underwent at least 1 week of acclimation to their new environment. They were kept in groups of 8–9 in standard transparent cages equipped with environmental enrichment. The mice had unlimited access to standard rodent chow and water. Maintaining consistent conditions, they were kept on a 12 h light/dark cycle (lights on at 6:00 a.m.), at 21–24 °C, 45–65% humidity, with regulated air circulation. All procedures for animal handling and care complied strictly with Directive 2010/63/EU of the European Parliament and the Council of 22 September 2010 [60], as well as the Polish Act of 15 January 2015 on the protection of animals used for scientific or educational purposes. The study protocol was approved by the Local Ethics Committee for Animal Experiments in Lublin, Poland (Approval No. 17/2023, issued on 30 January 2023).

#### 4.3.2. Study Design

In animal studies of acute and chronic asthma, mice are frequently used as test subjects. For this research, mice were divided into ten groups, each comprising 8 to 9 mice. The experiment included five groups with acute asthma induction and five with chronic asthma induction. To induce both asthma types, a raw fungal extract and ovalbumin, as a positive control, were used. Control group mice received either an adjuvant (Imject^®^Alum, Thermo Fisher Scientific, Rockford, IL, USA) or PBS.

#### 4.3.3. Acute and Chronic Asthma Model

**Figure 5:**
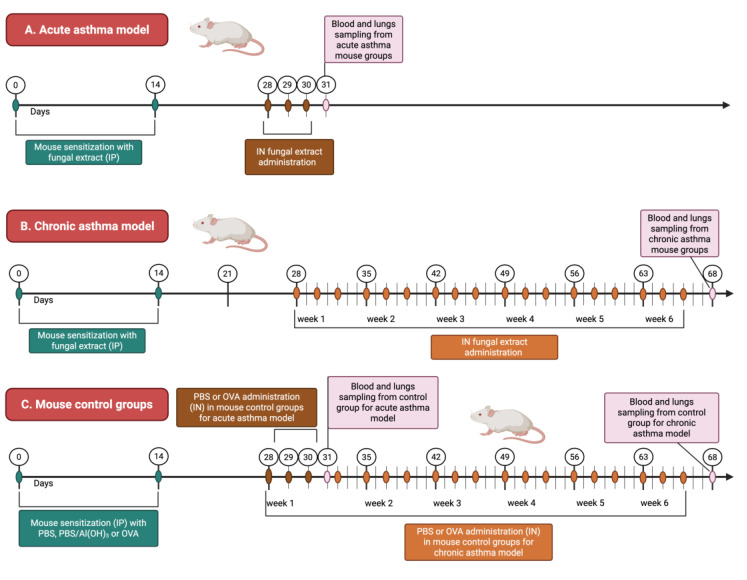
Chronological sequence illustrating the procedure for inducing and triggering acute (**A**) and chronic asthma (**B**) models in the experimental mouse groups in comparison with control groups (**C**) (created with BioRender.com, accessed on 24 September 2025).

Modified methods from Kim et al. [25] and Daubeuf et al. [26] were employed to establish both acute and chronic asthma models. Mice were sensitized intraperitoneally (IP) on days 0 and 14 with ovalbumin (OVA) [(40–500 μg/kg) in PBS/Al(OH)_3_ (1:1)] (Imject^®^Alum, Thermo Fischer Scientific) [23,24,25,26,27,28] or crude fungal extracts (400 μg of protein/kg) in PBS. The dose of 400 μg protein/kg was selected based on our previous in vitro work [23] and on published fungal asthma models using comparable extract doses that induce robust, yet non-toxic, allergic airway inflammation [26,27,28]. From day 21 onward, animals received intranasal (IN) challenges according to the schedule in Table 1. In the acute asthma model, mice were challenged on days 28, 29, and 30. In the chronic asthma model, mice were challenged three times per week for 6 weeks, starting on day 28, with each challenge containing 50 μg protein in 50 μL PBS per mouse. Control groups received alum/PBS only. Figure 1 shows the study timeline for inducing and triggering acute asthma (Figure 5A) and chronic asthma (Figure 5B), as well as the control groups (Figure 5C). For IN administration, mice were placed in a small, clear container and anesthetized with 2.5% isoflurane from a rodent anesthesia system (SomnoSuite, Kent Scientific, Torrington, CT, USA). Once anesthetized, the animals were held upright, and 20 μL of PBS, OVA, or fungal extract was delivered into the nostril using a micropipette. To ensure even distribution of the liquid in the airways, the animals remained upright for 30 s. For the acute asthma model (and appropriate control), biological samples were collected on day 31, while for the chronic asthma model, on day 68 of the experiment. The experiment was performed once, in accordance with ethical standards and using the minimum number of animals necessary for statistically valid results.

#### 4.3.4. Tissue Collection

Blood and lung samples were collected from mice in both the acute and chronic asthma groups 24 h after their final exposure, specifically on days 31 and 68, as shown in Figure 1. The animals were euthanized by decapitation. Trunk blood was gathered in sterile Eppendorf tubes without using an anticoagulant, and a single drop was used to create a blood smear on a glass slide. The blood was kept at 4 °C for 24 h, then centrifuged at 314× *g* for 5 min at room temperature (RT) after clotting. The sera were extracted and stored at −80 °C for later analysis. After decapitation, the lungs were extracted from the thoracic cavity, rinsed with ice-cold PBS, and immersed in 10% neutral buffered formalin (Sigma-Aldrich, St. Louis, MO, USA). Further experiments involving the biological materials (lungs and blood) were carried out in the Department of Virology and Immunology at Maria Curie-Skłodowska University in Lublin, Poland, in a BSL-2 type laboratory.

#### 4.3.5. Blood Smear Staining

After drying, the smears were stained using the May-Grünwald-Giemsa method. Each smear was treated with 1 mL of May-Grünwald stain solution (Sigma-Aldrich) for 3 min. Next, 1 mL of distilled water was added to each slide, the mixture was gently mixed with the stain, and the slides were left for an additional minute. The smears were then briefly washed with distilled water to remove excess stain. Before the next step, a 1:20 dilution of Giemsa stain (Sigma-Aldrich) was prepared with distilled water. About 1.5 mL of this diluted stain was applied to each slide and left for 15 min. The slides were then quickly rinsed and left to dry. In the stained samples, leukocytes—including lymphocytes, granulocytes, eosinophils, and monocytes—were counted under an Olympus BX53 light microscope (Olympus, Münster, Germany) at 100× magnification. A hematological counter was used to count 200 cells. Results were expressed as percentages of different leukocyte groups.

#### 4.3.6. Histological Analysis

Histological analysis of the lungs, focusing on eosinophil infiltration and airway remodeling, was performed at the Department of Clinical Pathomorphology, Medical University of Lublin. The left lung was fixed in formalin, embedded in paraffin, and sectioned at 3–5 μm thickness. These sections underwent hematoxylin-eosin (H&E) staining (Sigma-Aldrich) according to standard protocol [32,33]. Staining involved applying 500 mL of hematoxylin for 2 min at room temperature, followed by rinsing with tap water for 2 min. Next, 500 mL of eosin was applied for 1 min, and rinsed again. The stained sections were analyzed to assess the thickness of the airway epithelium and goblet cell lining, as well as eosinophilic infiltration in control and treated mice after allergen challenge. All samples were scored blindly, photographed, and analyzed using an Olympus BX40 microscope with a U-TV0.63XC digital camera (Olympus, Tokyo, Japan) and Olympus cellSens Standard Software (version 1.13), capturing images at ×4 or ×10 magnification.

This experiment was part of a larger study; therefore, the negative control (PBS), adjuvant control, and positive control (OVA) groups shared with those reported in our previous work [34]. This approach was used to reduce the number of animals, in accordance with the 3Rs principles and the Ethics Committee’s recommendations.

#### 4.3.7. Measurement of Cytokine Levels

Using ELISA (Invitrogen, Carlsbad, CA, USA), the levels of proinflammatory cytokines were measured in the mouse sera according to the manufacturer’s instructions. The proteins analyzed included interleukin-4 (IL-4), interleukin-5 (IL-5), interleukin-13 (IL-13), TNF-α, and TGF-β. The detection limits for these proteins were 2 pg/mL, 3.3 pg/mL, 2.8 pg/mL, 3.7 pg/mL, and 7.8 pg/mL, respectively.

#### 4.3.8. Determination of Specific IgE Levels in Mouse Sera

The detection of specific IgE antibodies was carried out using methods established previously by the authors [35]. In summary, MaxiSorp immunoplates (Nunc, Roskilde, Denmark) were incubated overnight at 4 °C with 100 μL of fungal crude extracts or ovalbumin at 1 μg/mL. After washing with PBS-T (0.05% Tween 20 in PBS, pH 7.4), the wells were blocked with 300 μL of PBS containing 3% BSA (Sigma) at 37 °C for 40 min. Following removal of the supernatant and three washes with PBS-T, 100 μL of serially diluted serum samples were added to duplicate wells. The sera were diluted in PBS-T in a 1:2 serial dilution series (ranging from 1:64 to 1:65,536) against the fungal antigen for titration. Wells coated with BSA served as blanks, whereas wells containing only fungal antigens were used as controls for nonspecific antibody binding. The plates were then incubated at room temperature for 2 h and washed three times with PBS-T. Subsequently, 100 μL per well of appropriately diluted secondary anti-mouse antibodies conjugated to alkaline phosphatase were added to detect mouse IgE. Goat anti-mouse IgE antibodies were obtained from Abcam (ab19967). After 2 h of incubation at room temperature and three washes with PBS-T, 100 μL of the alkaline phosphatase substrate (SigmaFast p-Nitrophenyl phosphate) was added to each well and incubated for 30 min at room temperature. The reaction was then halted with 100 μL of 1 M NaOH, and absorbance at 405 nm was measured using a VICTOR X4 Multilabel Plate Reader (PerkinElmer, Waltham, MA, USA). Specific IgE titers were determined as the reciprocal of the highest serum dilution that produced a mean absorbance of 0.1.

### 4.4. Statistical Analyses

The Kolmogorov–Smirnov test was conducted to determine the data distribution. Based on these results, either a parametric (one-way ANOVA with Tukey post hoc) or a nonparametric (Kruskal–Wallis with Dunn’s multiple comparisons) test was used to evaluate differences between the control group and other factors across multiple samples. For correlation analysis, the Spearman rank correlation coefficient was applied. Results were considered statistically significant at a *p*-value below 0.05. Data analysis was performed using STATISTICA software version 12 (StatSoft, Inc., Tulsa, OK, USA) and GraphPad Prism 8.4.3 (GraphPad Software, San Diego, CA, USA).

## 5. Conclusions

This study explores two previously uncharacterized phytopathogenic fungi, *T. pruni-spinosae* and *P. rubi-idaei.* When introduced to a mouse model of both acute and chronic asthma, these fungi triggered strong inflammatory and asthma-like responses, surpassing the effects caused by ovalbumin, a common allergen. The results were supported by increased inflammatory cytokines, specific IgE antibodies, and visible inflammatory damage in the mice’s lungs after intranasal exposure to the fungal allergens. Our research broadens the range of known fungal allergens, which could be crucial in the diagnosis of respiratory allergies. Additionally, the host plants for these fungi are frequently found in human environments, such as fruit orchards (*Prunus domestica*) and various wild and cultivated areas (*Rubus idaeus*). Further studies are needed to assess whether people with undiagnosed asthma might exhibit allergic reactions to these fungi.

A limitation of our study is that it utilizes basic microfungal extracts, which require standardization to identify key active components like proteins, fatty acids, or their complexes. Future research should also explore a more physiologically relevant sensitization pathway to better assess the plant microfungi’s pro-allergenic potential. Another limitation of this study is that lung histopathological changes were evaluated qualitatively, without the use of a blinded semi-quantitative scoring system or morphometric measurements (e.g., inflammation scores, eosinophil counts per field, or airway wall thickness), which may limit the robustness of between-group comparisons.

## Figures and Tables

**Table 1:** Procedure for inducing and triggering acute and chronic asthma in the examined mouse populations.

Group	Sensitization	Challenge	
	Day 0 and 14 (IP)	Acute Asthma	Chronic Asthma
		Day 28, 29, and 30 (IN Under General Anesthesia)	3× per Week for 6 Weeks Starting from Day 28 (IN Under General Anesthesia)
1. Negative control (PBS)	PBS	20 μL PBS/mouse	20 μL PBS/mouse
2. Adjuvant control (Al(OH)_3_)	PBS/Al(OH)_3_ (1:1)	20 μL PBS/mouse	20 μL PBS/mouse
3. Positive control (OVA)	OVA (40–500 μg/kg) in PBS/Al(OH)_3_ (1:1)	OVA (conc. 1 mg of protein/mL)—20 μL/mouse	OVA (conc. 1 mg of protein/mL)—20 μL/mouse
4. *T. pruni-spinosae*	Fungal extract (400 μg of protein/kg) in PBS	Fungal extract (conc. 1 mg of protein/mL)—20 μL/mouse	Fungal extract (conc. 1 mg of protein/mL)—20 μL/mouse
5. *P. rubi-idaei*			

OVA—ovalbumin, PBS—phosphate-buffered saline, Al(OH)_3_—adjuvant.

## Data Availability

The data that support the findings of this study are openly available in the Zenodo repository under the DOI: https://zenodo.org/records/17617796, accessed on 15 November 2025.
